# O.3.2-9 Sustaining activity with arthritis (SAWA) following an Arthritis Ireland Be active with arthritis (BAWA) exercise programme

**DOI:** 10.1093/eurpub/ckad133.154

**Published:** 2023-09-11

**Authors:** Aoife Stephenson, Helen French, David French, Deirdre Hurley, Aisling Walsh, Oliver Fitzgerald, Kathleen Bennett, Maria Stokes, Mick Thacker, Grainne O'Leary, Claire Kinneavy, Suzanne McDonough

**Affiliations:** Royal College of Surgeons in Ireland, Ireland; Royal College of Surgeons in Ireland, Ireland; University of Manchester; University College Dublin; Royal College of Surgeons in Ireland, Ireland; University College Dublin; Royal College of Surgeons in Ireland, Ireland; University of Southampton; Royal College of Surgeons in Ireland, Ireland; Arthritis Ireland; Arthritis Ireland; Royal College of Surgeons in Ireland, Ireland

## Abstract

**Purpose:**

Despite evidence demonstrating the health benefits of physical activity (PA), people with arthritis often do not meet recommended PA levels. Whilst various exercise programmes support these people to be active such as the ‘Be Active with Arthritis’ (BAWA) programme from Arthritis Ireland, most individuals reduce their PA after completion of prescribed programmes.

**Aim:**

To co-develop and pilot test a PA maintenance intervention for those who have participated in BAWA.

**Methods:**

This is a mixed methods project with four phases.

**Phase 1:**

An evidence synthesis will evaluate effectiveness of interventions for PA maintenance in people with arthritis and extract potential intervention components.

**Phase 2:**

An observational analysis of BAWA programmes will be undertaken to understand whether behaviour change components that might support PA maintenance are being currently used. This is followed by focus groups with 1) people with arthritis, 2) their carers, 3) the physiotherapists delivering the classes and 4) staff from Arthritis Ireland, to identify support needs and preference for development of a long-term PA programme.

**Phase 3:**

Data from Phases 1-2 will be triangulated and fed into participatory workshops, to co-develop an intervention. People with arthritis and healthcare/exercise professionals will be invited to these workshops.

**Phase 4:**

The PA programme, developed in Phase 3, will be piloted using a pre-post evaluation design with 25 people with arthritis in three separate waves. Interviews with programme attendees will explore facilitators of sustaining PA and identify implementation barriers. Public patient involvement will be embedded throughout this research with the formation of a PPI panel and an embedded Patient Researcher, to ensure that patients’ and carers’ lived expertise informs the various phases and final PA intervention.

**Results:**

Given the early stage of this piece of research, we would welcome feedback from the HEPA community on our plans, and gain insights from similar studies.

**Conclusion:**

By enhancing and developing service provision modalities, people with arthritis can remain physically active for as long as possible, improving quality of life, reducing pain, disability, co-morbidities and multi-service use.

**Funding:**

This project is jointly funded by the Irish Health Research Board and Arthritis Ireland.

